# Russo's Methylene Blue Reaction

**Published:** 1912-04-27

**Authors:** T. Thomson Rankin

**Affiliations:** A.M.O. City Fever Hospitals, Leeds.


### OUR FEVER HOSPITALS AND THEIR WORK.

(We shall be glad to have contributions for this section covering both medical and administrative points)

### Russo's Methylene Blue Reaction.

By T. THOMSON RANKIN, M.D., Senior A.M.O. City Fever Hospitals, Leeds.

Some years ago Russo introduced as a test for typhoid fever a watery solution of methylene blue (1-1,000) and advocated it as being simpler and of greater diagnostic value than Ehrlich's diazo reaction. This test does not seem to have been largely adopted, and the following remarks may be of some value. Rolleston (Metropolitan Asylums Board Report, 1905) reported favourably upon it and gave his experiences in a number of cases. He said it was more persistent than the diazo reaction, more often present in relapses than Ehrlich's test, and was of as great diagnostic value as the diazo reaction. He also considered its disappearance as corresponding more closely with lysis than the disappearance of the diazo reaction. Ker ("Manual of Infectious Diseases, 1910") does not seem to think much of it, and considers it inferior to Ehrlich's reaction.

### Rationale.

Four drops of a 1-1,000 watery solution of methylene blue are added to four or five cubic centimetres of urine in a test tube and the resulting mixture is shaken. If the reaction is positive a uniform mint or emerald-green colour is obtained. If, on the other hand, the reaction is negative a light green or a blue colour results. Before applying the test it is advisable to filter the urine if it is turbid from phosphates or any other cause. A variety of colours, or rather tints, occur between the emerald green and the blue, and it is a good plan to have a colour chart. This can easily be made by having the various colours painted in a row on a stout piece of cardboard. If the urine testing is done by the nursing staff this chart enables one to secure uniform results at all times and under all conditions. For convenience and uniformity in recording results, the following symbols are useful: R ++ indicating a deep green; R + a distinct green; R a green; R — a light green; and R — — a blue, colour. These symbols should also be attached to the colour chart.

This test has been applied in this hospital for a year or two, and the following observations are based on the cases certified to be suffering from typhoid fever during the past six or seven months. In most instances it has been continued for some time after convalescence has been established. A number of cases of scarlet fever were also examined, and the results will be given.

### Typhoid Fever.

All the cases diagnosed as enteric fever were confirmed by Widal's serum reaction. The diseases not confirmed as typhoid included copræmia, pneumonia, empyema, gastro-enteritis, rheumatism, endocarditis, peritonitis, suppuration of the shoulder-joint, and various forms of tuberculosis. Of these the following gave a positive reaction: copræmia, pneumonia, empyema, rheumatism, gastro-enteritis, peritonitis, suppuration of the shoulder-joint, and phthisis pulmonalis. All the cases of pneumonia and empyema and a proportion of the others gave the positive reaction.

The typhoid cases will be considered under various headings:—

(1) The day of disease on admission is an important factor. The later the case is admitted the greater the chance of a negative result. Few cases came under observation during the first week of illness, a fair number during the second week, and the majority at a later stage. About 11 per cent. of cases failed to give a positive result, and most of these were admitted at a late stage of the disease. A positive diazo reaction was present in 81 per cent. and a negative diazo in 19 per cent. of those presenting a positive Russo. In a few instances a negative Russo and a positive diazo reaction occurred. This shows that Russo's test occurs in a greater number of the cases admitted to hospital than Ehrlich's reaction.

(2) As regards the persistence of the diazo reaction, it was found that Russo's test was more persistent in the majority of cases. This was evident in at least 50 per cent. of cases. Ten per cent. of the cases had a more persistent diazo reaction. These facts tend to prove the assertion that Russo's test is more persistent than the diazo reaction.

(3) Rolleston, in his article, states that the disappearance of Russo's test corresponds more closely with lysis than the disappearance of the diazo reaction. In the series of cases under consideration, it was not found that lysis corresponded with the disappearance of either reaction. A disappearance of the Russo test corresponded more frequently with a continued normal temperature than did the disappearance of Ehrlich's reaction. In some cases both reactions continued positive for some days after a normal temperature was recorded. No complications or relapses occurred in these cases. There was no evidence to show that the disappearance of the reaction was a surer sign of convalescence than the disappearance of the diazo reaction beyond what is stated above.

(4) The intensity of the reaction varied at different stages of the disease and according to the severity of the attack. As the acute stage subsided and convalescence approached, the intensity diminished, and towards the end a positive result was obtained between negative results. These are also true of the diazo reaction. Fatal cases showed a most intense reaction to the end except in one instance, when a negative result obtained for a few days and was succeeded by an intense positive reaction just before death. Severe cases presented both a positive Russo and a positive Ehrlich, and both reactions were intense and persistent. This also occurred in cases complicated by hæmorrhage.

(5) The presence of the reaction after it had been negative did not help in any way to warn one of impending complications or relapses. In some cases a positive result was obtained after the complication had occurred. Thus it will be seen that Russo's test was of no use as far as the prognosis of complications or relapses was concerned.

(6) Rolleston says that a positive Russo is more common in relapses than a positive diazo reaction. This was not confirmed; a positive Ehrlich was as frequent as a positive Russo. Neither occurred earlier than the third day of the relapse.

(7) The earliest appearance of the reaction was on the fourth day, and it was obtained as far on as the fifth week. It is said to occur as early as the second day (Rolleston).

### Other Diseases.

A number of cases of scarlet fever were examined at regular intervals for varying periods for the presence or absence of this reaction. In addition, Ehrlich's diazo reaction was also employed. Russo's test was positive in 35 per cent. of the cases, and Ehrlich's in the same number. The former was positive on more occasions than the latter. In no instance was there any doubt as to the diagnosis of the case, and there was no co-existing disease at any time. A positive result did not occur on any particular day and was not related to any rise of temperature, any complication, or any co-existing disease. It was not of any diagnostic or prognostic significance.

In addition to the diseases mentioned above, a positive result was obtained in measles and erysipelas. This proves that Russo's reaction is not pathognomonic of typhoid fever.

### Summary.

Russo's methylene blue reaction occurs frequently in typhoid fever, pneumonia, measles, and empyema, occasionally in scarlet fever, and at times in various other diseases.

It persists longer than the diazo reaction in enteric fever, and occurs in a greater number of cases.

It is of some diagnostic value in enteric fever.

Its disappearance does not correspond more closely with lysis than the disappearance of the diazo reaction.

The intensity of the reaction is in some instances an index of the severity of the case.

It is of no value as regards warning one of impending complications or relapses.

It does not occur more frequently in relapses than the diazo reaction.

